# Eye care service utilization and associated factors among adults in Debre Berhan Town, North Shewa, Ethiopia, 2023

**DOI:** 10.3389/fpubh.2024.1440357

**Published:** 2024-09-19

**Authors:** Matiyas Mamo Bekele, Abebech Fikade Shumye, Melkamu Temeselew Tegegn

**Affiliations:** Department of Optometry, College of Medicine and Health Sciences, Comprehensive Specialized Hospital, University of Gondar, Gondar, Ethiopia

**Keywords:** proportion, eye care service utilization, Debre Birhan town, Ethiopia

## Abstract

**Introduction:**

Globally, the utilization of eye care services varies widely, ranging from 18 to 90%. Low utilization of eye care services can lead to delayed detection of vision problems. However, there are limited data on the proportion of eye care service utilization and its associated factors in Ethiopia at the community level.

**Objective:**

The objective of this study was to determine the proportion of eye care service utilization and its associated factors among adults in Debre Birhan town, North Shewa, Ethiopia.

**Methods:**

Using a multistage sampling method, a community-based cross-sectional study was conducted on 960 adults in Debre Birhan town from 8 May to 8 June 2023. Binary logistic regression was performed. Variables with a *p*-value of less than 0.05 were considered significant.

**Results:**

A total of 940 study participants participated, resulting in a response rate of 97.9%. The mean age of the participants was 54.67 years (SD ± 8.69). The proportion of good-level eye care service utilization was found to be 32.98% [95% confidence interval (CI): 29.97, 35.99]. Factors positively associated with good-level eye care service utilization included older age [adjusted odds ratio (AOR) = 1.58, 95%CI: 1.00, 2.51], higher educational status (AOR = 2.25, 95%CI: 1.25, 4.06), high family monthly income (AOR = 8.70, 95%CI: 4.99,15.18), awareness of regular eye checkups (AOR = 1.77, 95%CI: 1.15, 2.73), a history of eye disease (AOR = 2.57, 95%CI: 1.77,3.74), health insurance (AOR = 1.99, 95% CI: 1.34, 2.95), and history of spectacle use (AOR = 1.94, 95%CI:1.34, 2.80).

**Conclusion:**

The study revealed that the proportion of good-level eye care service utilization was low. Older age, higher educational status, high family monthly income, awareness of regular eye checkups, a history of eye disease, health insurance, and history of spectacle use were significantly associated with a good level of eye care service utilization.

## 1 Introduction

Eye care service utilization refers to the use of eye care services by individuals for the prevention and treatment of eye problems, promotion of eye health, and acquisition of information about their eye health status and prognosis over a specific period (1).

Globally, the utilization of eye care services varies widely, ranging from 18 to 90% (2–6). High proportions (60–90%) of eye care service utilization have been reported in high-income nations such as the United States, Australia, and Canada (2–4). In contrast, low, lower-middle, and upper-middle-income nations have reported utilization rates of 22, 24, and 37%, respectively (5). Similarly, a study conducted in Ethiopia reported a utilization rate of 23.8% (6).

A low proportion of eye care service utilization can delay the early detection of vision problems, leading to visual impairment. It is estimated that at least 2.2 billion people around the world suffer from visual impairment (7). The burden of visual impairment is not evenly distributed across the globe, with the least developed nations contributing the largest portion. Currently, 90% of cases of vision loss occur in middle- and low-income countries due to limited access to eye care services (8). In Africa, it is estimated that at least 26.3 million people suffer from visual impairment (9). The prevalence of visual impairment in Ethiopia was found to be 6.24% (10).

Visual impairment can lead to increased unemployment rates, reduced productivity, higher medical expenses, limitations in daily living activities, and decreased social participation, ultimately resulting in a reduced quality of life (11).

Research indicates that the main factors associated with eye care service utilization are socioeconomic, clinical, and personal factors, as well as a lack of access to eye care services (12).

The American Academy of Ophthalmology recommends practical guidelines and periodic eye examinations based on age and associated risk factors (13). Regular eye examinations for asymptomatic patients can help detect a significant number of new eye conditions and lead to changes in care (14).

Eye care service utilization has become a major public health concern in developing countries, necessitating significant attention. Despite this, in Ethiopia, only three studies have been conducted on eye care services (6, 15, 16), with two of them focusing on southern Ethiopia (6, 16). However, there is a lack of evidence regarding eye care service utilization in northeast Ethiopia, including the study area. This study aimed to fill this research gap by assessing the proportions of eye care service utilization and identifying associated factors among adults aged 40 years and above in northeast Ethiopia. In addition, the findings of this study will provide valuable information to plan and prioritize eye health programs for the early detection and treatment of eye diseases. Finally, this study aimed to determine the proportion of eye care service utilization and its associated factors among adults living in Debre Birhan town, North Shewa, Ethiopia.

## 2 Materials and methods

### 2.1 Study design, period, and area

A community-based cross-sectional study was carried out in Debre Birhan town, North Shewa, Ethiopia, from 8 May to 8 June 2023. The town is situated 130 km from Addis Ababa, the capital city of Ethiopia, and 688 km from Bahir Dar, the capital city of Amhara. The total population is 88,375, consisting of 39,961 male individuals and 48,414 female individuals, with 56,914 being adults aged 40 years and above. The town has nine administrative regions (kebeles) and is home to a tertiary eye care center staffed with ophthalmologists, optometrists, ophthalmic nurses, and general nurses, providing services to over 3 million people in the catchment area. Additionally, there is a private eye clinic and more than three optical workshops in the town.

### 2.2 Study population and eligibility criteria

All adults aged 40 years or older who had been residing in Debre Birhan town for at least 6 months during the data collection period were eligible to participate in the study. However, individuals who were unable to complete the questionnaire due to severe illness or mental impairment were not included.

### 2.3 Sample size determination

The sample size was determined using a single proportion formula that *n* = $\frac{\left(Z\;\alpha /₂\right)2x\;P\;\left(1-P\right)}{d2}$ with the following consideration (*n* = sample size, Z = the value of the z statistic at 95% confidence level = 1.96, P = the expected proportion of eye care service utilization was 23.8%, based on a previous study conducted in Hawassa, Ethiopia (6), and *d* = margin of error of 4%). According to this, the calculated sample size was 436. Considering a design effect of 2 and a 10% non-response rate, the final planned sample size was 960.

### 2.4 Sampling technique and procedures

In Debre Birhan town, there are nine kebeles. To ensure a representative sample, a multistage sampling procedure was used. Initially, four kebeles out of the nine were selected through a lottery method. The sample size was allocated proportionally to each selected kebele. A systematic random sampling method was then used to select households, with a constant interval of 11. This interval was determined by dividing the total number of households in the selected kebeles (10,519) by the sample size (960). To choose the first household, a number between 1 and 11 was randomly selected, and every 11th household was subsequently included in the sample. If more than one eligible adult aged 40 years or older was found in the selected household, study participants were recruited using a lottery method. If the eligible person could not be located during data collection, the household was revisited twice. If there was no eligible person in the selected household who met the inclusion criteria, an immediate neighboring household was included in the survey.

### 2.5 Operational definition

#### 2.5.1 Eye care service utilization

If an individual has visited an eye care center for an eye examination at least once within the past 2 years, they are considered to have good eye care service utilization. On the other hand, if an individual has not visited an eye care center for an eye examination within the past 2 years up until the data collection period, they are considered to have poor eye care service utilization (13).

#### 2.5.2 Has escort

Participants who have someone to help them visit eye care service centers for their eye checkups.

### 2.6 Data collection tool and procedure

The data were collected by five well-trained optometrists using a pretested structured questionnaire through face-to-face interviewers, which means the optometrist asked the questions to the study participants and recorded their responses for each question.

The questionnaire was adapted from previous studies on similar topics (6, 17). It was originally prepared in English and then translated into Amharic for data collection and subsequently translated back to English by local language translators during data entry to maintain consistency and accuracy. The questionnaire included demographic data, information on eye care services, behavioral factors, and medical and clinical conditions.

### 2.7 Data processing and analysis

After ensuring the completeness and consistency of the data, the information was entered into Kobo Collect version 2021.4.4 and then exported to Stata version 14 for analysis. Both descriptive and analytical statistics were performed. Multicollinearity was checked using variance inflation factor (VIF) and tolerance. A bivariable logistic regression was conducted, followed by a multivariable binary logistic regression to identify potential predictors of eye care service utilization. The strength of the association between predictors and outcome variables was indicated using an AOR with a 95% CI. The model’s fitness was verified through the Hosmer and Lemeshow goodness-of-fit test. Variable with a *p*-value of 0.05 or less in the multivariable binary logistic regression was considered statistically significant.

## 3 Results

### 3.1 Socio-demographic characteristics of the study participants

In this study, 940 participants participated, achieving a response rate of 97.9%. The average age of the participants was 54.67 years (SD ± 8.69). Out of the 940 participants, 673 (71.60%) were male, 761 (80.96%) were married, and 390 (41.49%) were enrolled in higher education (college and above) (Table 1).

**Table 1:** Socio-demographic characteristics of the study participants in Debre Birhan town, North Shewa, Ethiopia, 2023 (*n* = 940).

Variables	Categories	Frequency	Percentage
Age(in year)	40–54	427	45.43
	55–64	315	33.51
	≥65	198	21.06
Sex	Male	673	71.60
	Female	267	28.40
Marital status	Not married	108	11.49
	Married	761	80.96
	Divorced	45	4.79
	Widowed	26	2.77
Educational status	Unable to read and write	125	13.30
	Able to read and write	210	22.34
	Primary school	80	8.51
	Secondary School	135	14.36
	College and above	390	41.49
Occupational status	Merchant	235	25.00
	Government employed	416	44.26
	Housewife	108	11.49
	Private employed	141	15.00
	Unemployed	40	4.26
Family monthly income (Ethiopian birr)	500–2000	310	32.98
	2001–4,000	255	27.13
	4,001–5,999	201	21.38
	≥6,000	174	18.51

### 3.2 Medical and eye care service information-related factors

Out of 940 respondents, 49.26% reported having a history of eye problems or disease. Among them, 143 people, or 30.8%, stated that their eye problem affected their daily activities. However, only 630 (67.00%) participants had visited an eye care center for an eye examination in their lifetime (Table 2).

**Table 2:** Medical and eye care service information-related factors of study participants in Debre Birhan town, North Shewa, Ethiopia, 2023 (*n* = 940).

Variables	Categories	Frequency	Percent
History of eye disease	Yes	463	49.26
	No	477	50.74
Family history of eye disease	Yes	262	27.87
	No	678	72.13
Awareness about regular eye checkups	Yes	646	68.72
	No	294	31.28
Ever visited an eye care center for an eye checkup	Yes	630	67.00
	No	310	33.00
Reason for not visiting eye care service (*n* = 310)	No eye problem	130	41.93
	No information	100	32.26
	Financial problem	57	18.39
	I do not know the place	10	3.23
	Traditional medicine	13	4.19
Preferred place for eye check-up (*n* = 630)	Eye care clinic	203	32.22
	Hospital	170	26.98
	Health center	123	19.52
	Holy water	61	9.68
	No where	56	8.99
	Traditional medicine	17	2.61
Health insurance	Yes	312	33.19
	No	628	66.81
History of diabetes	Yes	75	7.98
	No	865	92.02
History of hypertension	Yes	16	1.70
	No	924	98.30

### 3.3 Proportion of eye care service utilization among study participants

The present study found that the good level of eye care service utilization was 32.98% (95% CI: 29.97, 35.99).

### 3.4 Factors associated with eye care service utilization

In a bivariable binary logistic regression analysis, several factors were found to be significantly associated with a good level of eye care service utilization. These factors include older age, higher educational status, increased family monthly income, awareness about regular eye checkups, history of eye disease, having health insurance, history of spectacle use, family history of eye disease, having an escort, history of diabetes mellitus, and history of hypertension. However, in the multivariable binary logistic regression analysis, only older age, higher educational status, increased family monthly income, awareness about regular eye checkups, history of eye disease, having health insurance, and history of spectacle use were found to be significantly associated with a good level of eye care service utilization.

This study revealed that the odds of a good level of eye care service utilization among participants aged ≥65 years were 1.58 times higher than those aged 40–54 years (AOR = 1.58, 95% CI: 1.00, 2.51).

Participants with a higher level of education (college and above) were 2.25 times more likely to have eye care service utilization than those who cannot read and write (AOR = 2.25, 95% CI: 1.25, 4.06).

Participants with a family monthly income of ≥6,000 Ethiopian birr were 8.70 times more likely to have eye care service utilization than participants with a family monthly income of 500–2000 Ethiopian birr (AOR = 8.70, 95% CI: 4.99, 15.18).

The odds of a good level of eye care service utilization among participants with a positive history of awareness of regular eye checkups were 1.77 times higher than those who had no positive history of awareness of regular eye checkups (AOR = 1.77, 95% CI: 1.15, 2.73). The odds of utilizing eye care services were 2.57 times (AOR = 2.57, 95% CI: 1.77, 3.74) higher for participants with a history of eye disease than their counterparts.

Participants who had health insurance were 1.99 times more likely to utilize eye care services than participants who did not have health insurance (AOR = 1.99, 95% CI: 1.34, 2.95). The odds of a good level of eye care service utilization in those participants with a history of spectacle use were 1.94 times higher than participants with no positive history of spectacle use (AOR = 1.94, 95% CI: 1.34, 2.80) (Table 3).

**Table 3:** Factors associated with eye care service utilization among adults in Debre Birhan Town, North Shewa, Ethiopia, 2023 (*n* = 940).

Variables	Eye care service utilization				
	Good	Poor	COR (95% CI)	AOR (95% CI)	*p*-value
Age (in years)					
40–54	92	335	1.00	1.00	
55–64	125	190	2.39 (1.73–3.30)	1.00 (0.65–1.53)	0.995
≥65	93	105	3.22 (2.24–4.63)	1.58 (1.00–2.51)	0.049
Sex					
Female	56	211	1.00	1.00	
Male	254	419	2.28 (1.63–3.18)	1.41 (0.92–2.15)	0.110
Educational status					
Unable to read and write	25	100	1.00	1.00	
Able to read and write	51	159	1.28 (0.74–2.20)	1.20 (0.64–2.27)	0.556
Primary school	14	66	0.84 (0.41–1.75)	1.09 (0.46–2.59)	0.830
Secondary School	52	83	2.50 (1.43–4.38)	2.26 (1.15–4.44)	0.017
College and above	168	222	3.02 (1.86–4.90)	2.25 (1.25–4.06)	0.007
Family monthly income (Ethiopian birr)					
500–2000	32	278	1.00	1.00	
2001–4,000	56	199	2.44 (1.52–3.91)	1.53 (0.90–2.60)	0.110
4,001–5,999	104	97	9.31 (5.88–14.73)	5.08 (2.98–8.66)	<0.0001
≥6,000	118	56	18.30 (11.27–29.72)	8.70 (4.99,15.18)	<0.0001
Awareness about regular eye checkups					
Yes	259	387	3.18 (2.26–4.48)	1.77 (1.15–2.73)	0.009
No	51	243	1.00		
History of eye disease					
Yes	222	241	4.07 (3.03–5.46)	2.57 (1.77–3.74)	<0.0001
No	88	389	1.00	1.00	
Health insurance					
Yes	176	136	4.77 (3.55–6.40)	1.99 (1.34–2.95)	0.001
No	134	494	1.00	1.00	
History of spectacle use					
Yes	166	163	3.30 (2.48–4.39)	1.94 (1.34–2.80)	<0.0001
No	144	467	1.00	1.00	
Family history of eye disease					
Yes	47	215	0.34 (0.24–0.49)	0.90 (0.56–1.44)	0.673
No	263	415	1.00	1.00	
Had escort					
Yes	166	275	1.48 (1.13–1.95)	0.87 (0.60–1.27)	0.493
No	144	355	1.00	1.00	
Diabetes					
Yes	24	51	0.95 (0.57–1.57)	1.07 (0.57–1.99)	0.828
No	286	579	1.00		
Hypertension					
Yes	14	13	2.24 (1.04–4.83)	1.74 (0.62–4.90)	0.288
No	296	617	1.00		

COR, Crude odd ratio; AOR, adjusted odd ratio, and CI, confidence interval.

## 4 Discussion

In this study, the proportion of individuals with a good level of eye care service utilization was 32.98% (95% CI: 29.97, 35.99), similar to the 32% reported in a study conducted in Gondar town, Ethiopia (17). This alignment may be attributed to similarities in the study populations, both focusing on older populations, as well as comparable study designs and ocular examination of participants.

However, the proportion of eye care service utilization in this study was higher than in the studies conducted in Hawassa City, Ethiopia (23.8%) (6), southern Ethiopia (29%) (16), Northwest Ethiopia (21%) (15), and Nigeria(18%) (18). The variation in eye care service utilization in different populations can be significantly influenced by socioeconomic factors such as age, education, occupational status, and income. The study conducted in Hawassa and Meskan, southern Ethiopia, highlighted that the educational status of participants was predominantly low, with fewer individuals having completed secondary education or higher. Additionally, there was a limited representation of government and non-government occupations among the study participants, and only a small number of participants were aged over 65 years. In contrast, the current study indicates that a larger proportion of participants had higher education levels, were employed in various occupations, and included older individuals, which correlates with increased awareness and utilization of eye care services. Furthermore, the study conducted in Nigeria included adults over 18 years old, while the current study specifically targeted individuals aged 40 and above. Older adults generally require more eye care due to age-related conditions such as cataracts, glaucoma, and macular degeneration. As a result, this demographic is more inclined to seek eye care, leading to higher utilization rates in studies that involve a larger proportion of older participants (6).

This study indicates that subjects aged ≥65 years had 1.58 times higher odds of good eye care service utilization than those participants aged 40–54 years. This finding is consistent with the studies conducted in Hawassa, Ethiopia (6), Southern Ethiopia (16), northwest Ethiopia (19), and Nigeria (18). This association may be attributed to the increased likelihood of developing age-related eye diseases such as cataracts, glaucoma, age-related macular degeneration, and presbyopia as individuals age. These conditions often require them to seek eye care services, leading to higher utilization rates among older adults (5).

Participants with a higher level of education (college and above) were 2.25 times more likely to have good eye care service utilization than participants who could not read and write. This finding corresponds with the study conducted in Nigeria (18). This association might be that higher education leads to increased utilization of eye care services due to better knowledge, access, and affordability. Educated individuals are often part of higher socioeconomic classes, allowing them better access to eye care services. Education also enhances awareness of the importance of regular eye checkups, leading to a proactive approach to eye health. Improving education levels can help increase access to critical eye care services, especially in underserved communities (6, 20).

The current study found that a good level of eye care service utilization was approximately 8.70 times more in individuals with a family monthly income of ≥6,000 Ethiopian birr than those participants with a family monthly income of 500–2000 Ethiopian birr. This finding is supported by the studies conducted in Hawassa, Ethiopia (6), and Nigeria (18). The association could be because higher income levels are generally linked to better access to healthcare services. People with more financial resources can afford the costs of eye care, including consultations, treatments, and follow-up appointments. This is especially important in places where healthcare costs can be very high. However, the high cost of eye care is a significant barrier to utilization, especially in rural and underserved communities. Improving affordability and access to eye care is crucial to increasing utilization for disadvantaged populations (5, 18, 21).

The odds of a good level of eye care service utilization among participants with a positive history of awareness of regular eye checkups were 1.77 times higher than those who had no positive history of awareness of regular eye checkups. This finding is in line with the studies conducted in Hawassa City, Ethiopia (6), and Southern Ethiopia (16). This association might be that awareness of regular eye checkups encourages individuals to seek eye care services, leading to early detection and management of eye conditions. Being informed about the significance of regular eye examinations increases the likelihood of individuals recognizing the need for preventive care, ultimately contributing to higher rates of eye care service utilization (18).

Individuals with a positive history of eye disease had 2.57 times higher odds of good eye care service utilization than their counterparts. This finding is consistent with the studies conducted in Hawassa, Ethiopia (6), Southern Ethiopia (16), and Nigeria (18). This association might be that people who have a history of eye disease are more likely to seek out eye care services than those who have not experienced eye health issues before. This is because they are more aware of the importance of regular eye checkups and are more susceptible to eye problems. Those who have had eye diseases in the past are more likely to prioritize their eye health and seek professional eye care services to prevent future complications or manage existing conditions effectively. This is why having a history of eye disease can influence the utilization of eye care services (15, 18).

The result of this study showed that participants who had health insurance were 1.99 times more likely to have good eye care service utilization than participants who did not have health insurance. This finding is consistent with the study conducted in Midda Welabo, Ethiopia (22). This association might be that health insurance can significantly reduce the costs of eye care services, making them more affordable and easier to access for beneficiaries. Research shows that Medicaid beneficiaries who have vision coverage are more likely to have an annual eye exam and less likely to report difficulties in affording eyeglasses. In summary, health insurance helps to remove financial barriers and improve access to both preventive and remedial eye care, which is particularly important for vulnerable populations (23).

Participants with a history of spectacle use had 1.94 times better odds of having good utilization of eye care services compared to participants who had no positive history of spectacle use. This finding is supported by the study conducted in Northwest Ethiopia (15). This association may be due to the fact people who wear spectacles are more likely to use eye care services. Individuals who wear might have existing eye problems or an increased understanding of the importance of eye care. Wearing spectacles can lead to increased awareness of eye health, establish relationships with eye care professionals, and encourage the utilization of services for preventive care. Furthermore, regular visits for eye examinations can help build lasting relationships with eye care professionals. These connections can motivate individuals to seek follow-up care, preventive services, and management of any developing eye conditions (24).

## 5 Limitations of the study

Since this is a cross-sectional study, it does not show the temporal relationship between predictors and eye care service utilization, and thus cannot determine the actual cause–effect relationship. In addition, the study did not include individuals living on the streets, as the sampling units were limited to households.

## 6 Conclusion

This study revealed that the proportion of good-level eye care service utilization was low. Factors such as older age, higher educational status, high family monthly income, awareness of regular eye checkups, a history of eye disease, health insurance, and history of spectacle use were significantly associated with a good level of eye care service utilization.

## Data Availability

The original contributions presented in the study are included in the article/supplementary material, further inquiries can be directed to the corresponding author.
