# Supplementary figures and images for: Eye care service utilization and associated factors among adults in Debre Berhan Town, North Shewa, Ethiopia, 2023
Source: Front Public Health. 2024 Sep 19;12:1440357. doi: 10.3389/fpubh.2024.1440357 (PMC11457573; doi:10.3389/fpubh.2024.1440357)

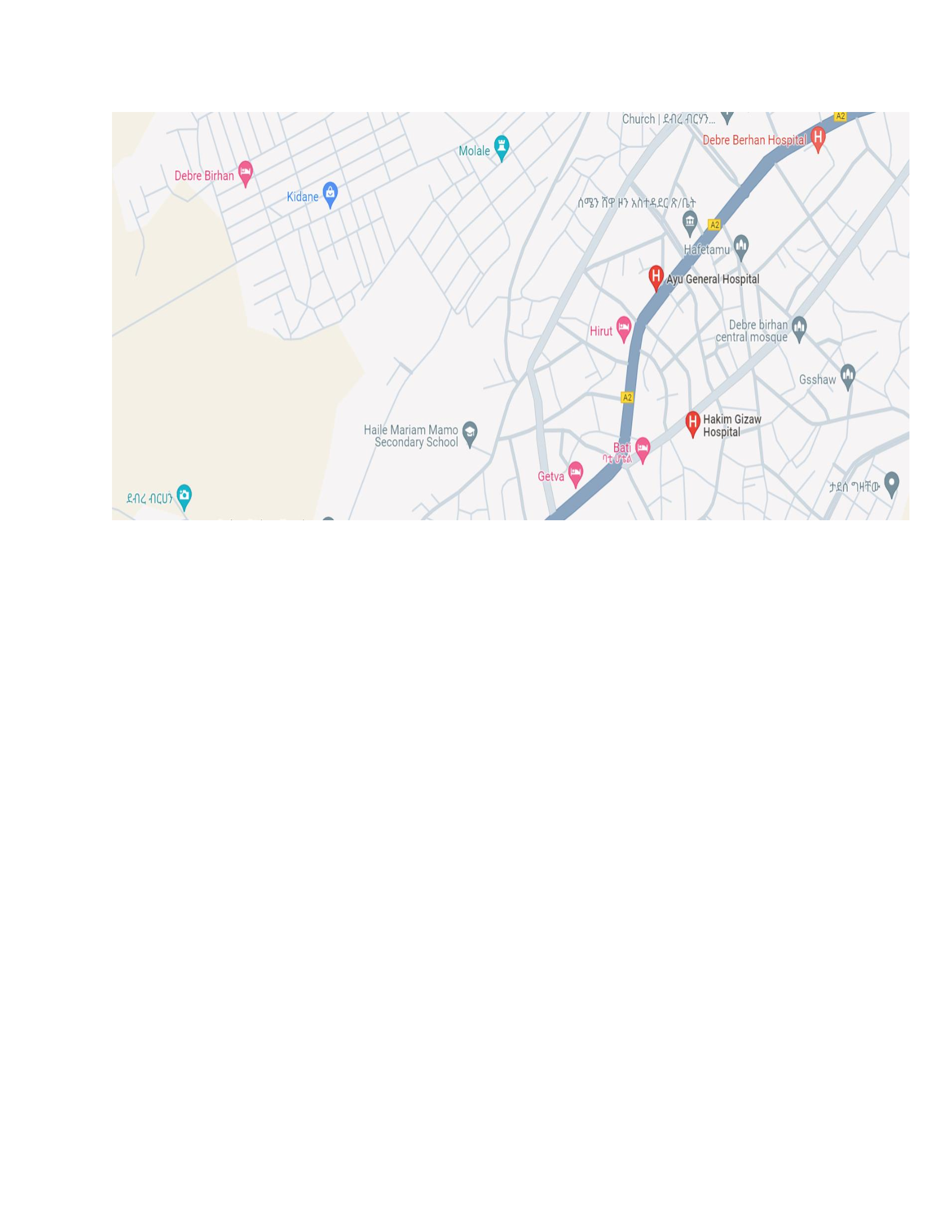

Supplement: Supplementary file 1 [file Image_1.TIFF]
